# Hypertriglyceridemia-Induced and Alcohol-Induced Acute Pancreatitis—A Severity Comparative Study

**DOI:** 10.3390/diagnostics15070882

**Published:** 2025-04-01

**Authors:** Monica Grigore, Daniel Vasile Balaban, Mariana Jinga, Florentina Ioniță-Radu, Raluca Simona Costache, Andrada Loredana Dumitru, Ionela Maniu, Mihaela Badea, Laura Gaman, Săndica Bucurică

**Affiliations:** 1 Department of Gastroenterology, Buzau County Emergency Hospital, 120140 Buzau, Romania; mona.grigore26@gmail.com; 2 Department of Internal Medicine and Gastroenterology, Carol Davila University of Medicine and Pharmacy, 020021 Bucharest, Romania; vasile.balaban@umfcd.ro (D.V.B.); mariana.jinga@umfcd.ro (M.J.); florentina.ionita-radu@umfcd.ro (F.I.-R.); raluca.costache@umfcd.ro (R.S.C.); 3 Department of Gastroenterology, University Emergency Central Military Hospital “Dr. Carol Davila, 010825 Bucharest, Romania; 4 Department of Mathematics and Informatics, Faculty of Sciences, Lucian Blaga University Sibiu, 550012 Sibiu, Romania; ionela.maniu@ulbsibiu.ro; 5 Research Team, Pediatric Clinical Hospital Sibiu, 550166 Sibiu, Romania; 6 Faculty of Medicine, Transilvania University of Brasov, 500019 Brasov, Romania; mihaela.badea@unitbv.ro; 7 Research Center for Fundamental Research and Prevention Strategies in Medicine, Research and Development Institute, Transilvania University of Brasov, 500484 Brasov, Romania; 8 Biochemistry Department, Carol Davila University of Medicine and Pharmacy, 020021 Bucharest, Romania; glauraelena@yahoo.com

**Keywords:** hypertriglyceridemia-induced pancreatitis, alcohol-induced pancreatitis, acute pancreatitis, Atlanta classification, acute pancreatitis severity

## Abstract

**Background:** Alcohol use and hypertriglyceridemia are the second and third common causes of acute pancreatitis after choledocholithiasis. Still, few studies directly compare the severity and outcomes of these two groups, which share pathophysiology pathways. **Methods:** In our study, we compared the biologic profile, severity according to the Atlanta classification and Balthazar index, intensive care unit admissions, and mortality between patients with hypertriglyceridemia-induced pancreatitis (HTGP) and alcohol-induced acute pancreatitis (AAP). A total of 78 patients were included in this study, 37.17% of which had HTGP, and 62.82% had AAP. **Results:** HTGP was more severe in terms of the Atlanta revised classification severity assessment (82.76% vs. 46%, *p* = 0.014), led to more extended hospitalizations (*p* = 0.024), and resulted in similar serum CRP levels among patients, with a significant difference regarding median serum fibrinogen values (739 vs. 563 mg/dL, *p* = 0.030) and necrotizing forms (24.13% vs. 10.20%). Hyponatremia was more significant in HTGP patients compared with AAP patients (130 vs. 137 mmol/L, *p* < 0.000). No differences were found in other inflammation indexes such as NLR (neutrophil count/lymphocyte count), PLR (platelet count/lymphocyte count), MLR (monocyte/lymphocyte count), SII (systemic immune-inflammation index), or SIRI (systemic inflammation response index). **Conclusions:** The pattern of acute pancreatitis is related to its etiology and may have different grades of severity. In our study, we found that hypertriglyceridemia-induced pancreatitis required twice as many admissions to the intensive care unit and was associated with lower serum sodium levels, and almost twice as many patients with HTGP had moderate or severe forms of acute pancreatitis compared to alcohol-induced pancreatitis cases.

## 1. Introduction

Acute pancreatitis is a condition characterized by acute inflammation of the pancreas. It is one of the most common gastrointestinal diseases that requires hospitalization. Recent studies have shown an increasing incidence rate, regardless of etiology [1]. Following the biliary lithiasis disease, alcohol use and hypertriglyceridemia are among the most common causes of acute pancreatitis. Both primary and secondary hypertriglyceridemia can cause acute pancreatitis, but secondary causes are more often involved in the etiology of hypertriglyceridemia-induced acute pancreatitis (HTPA) [2].

According to the Report of the American College of Cardiology/American Heart Association Task Force on Clinical Practice Guidelines, hypertriglyceridemia can be classified based on serum levels as mild (150–175 mg/dL), moderate (175–499 mg/dL), and severe (>500 mg/dL) [3].

A serum triglyceride level higher than 1000 mg/dL has been conventionally associated with acute pancreatitis, but the actual cutoff level is unknown, and individuals vary in this regard [4]. The risk of developing acute pancreatitis progressively increases with increasing levels of serum triglycerides [5]. Higher triglyceride levels are also associated with a higher risk of mortality, complications, and more severe forms of acute pancreatitis [6]. High levels of free fatty acids resulting from triglycerides hydrolyzed by lipase and blood viscosity are the most common mechanisms of HTGP [7]. The relationship between alcohol and plasma lipid profiles is strongly intertwined, with alcohol being one of the causes of secondary hypertriglyceridemia [8]. The effect of alcohol on serum lipids is variable. The pattern, the dose, the type, and the duration of alcohol intake, together with dietary factors, seem to influence how the lipid profile is altered [9,10,11]. While low to moderate alcohol consumption appears to result in increased levels of low-density lipoprotein cholesterol (LDL-C), low total cholesterol, and low triglycerides, heavy alcohol drinking causes significantly higher levels of serum triglycerides, total cholesterol, and LDL cholesterol, with lower levels of serum high-density lipoprotein cholesterol (HDL-C) [12]. The hyperlipidemic effect of alcohol is even more pronounced in obese patients [13].

Another possible mechanism implicated in hypertriglyceridemia regarding acute alcohol intake, especially in high doses, is the inhibition of lipoprotein lipase. Lipoprotein lipase is an enzyme localized on the vascular endothelium that plays a pivotal role in degrading serum triglycerides [14,15]. Alcohol toxicity in the pancreas is dose-dependent, and the risk of pancreatitis increases with alcohol intake. Acinar pancreatic cells metabolize alcohol in both oxidative and non-oxidative ways, generating several toxic metabolic products such as acetaldehyde, fatty acid ethyl esters (FAEEs), and reactive oxygen species. These products are the real culprits that damage acinar cells via various mechanisms. Alcohol can also induce the hyperviscosity of pancreatic juice, which can lead to precipitation and protein plug formation into the pancreatic ductular system. This can result in injury to the ductal epithelium and the obstruction of the pancreatic ducts [16,17]. Both alcohol and its metabolites activate pancreatic stellate cells. Once activated, they can convert into myofibroblast-like cells, which are pivotal in tissue regeneration in the early stages and later in fibrosis development [18]. Both etiologies, alcoholic and hypertriglyceridemia-induced pancreatitis, seem to be risk factors for recurrent bouts of pancreatitis. Still, few studies directly compare the severity and outcomes of these two groups [19].

While the precise mechanism by which gallstones induce pancreatitis remains unclear, multiple theories have been proposed for the pathogenesis of alcohol- and gallstone-induced pancreatitis, but none are universally accepted [20]. However, the pathophysiology of the induction of AP by gallstones and alcohol or hypertriglyceridemia appears to have distinct mechanisms, potentially influencing disease severity and complication rates [21,22]. Nonetheless, hypertriglyceridemia and alcohol-induced acute pancreatitis share common pathophysiological pathways such as cytotoxic acinar injury and oxidative stress [16,22,23,24,25].

This study aims to compare hypertriglyceridemia-induced acute pancreatitis (HTGP) and alcoholic acute pancreatitis (AAP) in terms of severity and outcomes.

## 2. Materials and Methods

### 2.1. Study Design and Patient Selection

We retrospectively reviewed the medical records of patients discharged from our center with acute pancreatitis between March 2018 and December 2022. The diagnosis of acute pancreatitis was based on revised Atlanta criteria [26,27] (Table 1). After the etiologies of acute pancreatitis were identified, patients with AAP and hypertriglyceridemia-induced acute pancreatitis (HTGP) were selected and included in the study.

Inclusion criteria are as follows:

- Patients with acute pancreatitis and hypertriglyceridemia with levels > 1000 mg/dL for Hypertriglyceridemia- induced acute pancreatitis;
- Patients with acute pancreatitis, with alcohol being mentioned as the primary cause in the medical records for alcohol-induced acute pancreatitis

Exclusion criteria are as follows:

- Patients with acute pancreatitis of etiologies other than HTGP and AAP;
- Patients with both concomitant etiologies or mixed causes;
- Patients with chronic pancreatitis;
- Patients with hypertriglyceridemia or alcohol abuse that had concomitant gallstones;
- Patients age < 18;
- Pregnant patients;
- Patients who stayed less than 1 day in the hospital (ex., discharged on request shortly after being admitted).

**Figure 1:**
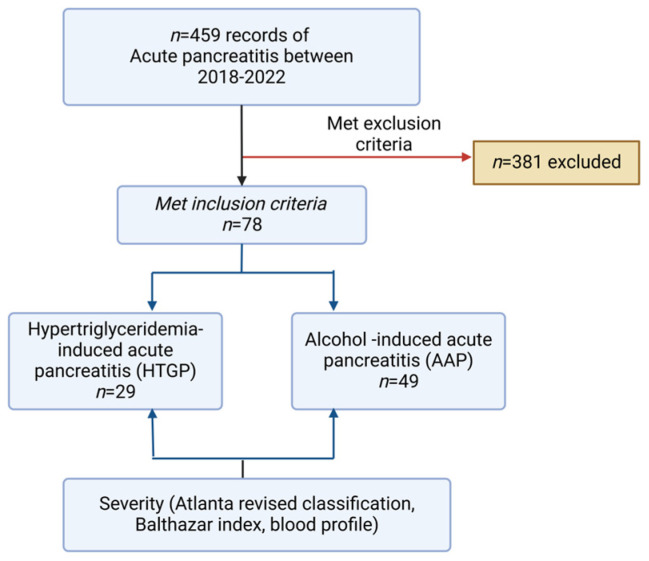
Study’s flow chart.

Hypertriglyceridemia was considered the etiology of acute pancreatitis if the patient met the diagnostic criteria for acute pancreatitis and their serum triglyceride levels were higher than 1000 mg/dL. Patients were considered to have alcoholic pancreatitis if there was no obvious etiology other than alcohol, and alcohol was mentioned in their medical records as the primary cause. Patients who were < 18 years or pregnant and who stayed less than one day at the hospital were excluded from this study. Patients with concomitant gallstones were also excluded from this study, even if the primary etiology was either alcohol or hypertriglyceridemia. Only the index admission was considered for patients with multiple admissions during the study period (Figure 1).

Multiple types of data were collected for this study, including demographic data (gender, age, comorbidities, smoking cigarettes, alcohol use, and high-fat diet), both localized and systemic factors influencing severity (Atlanta severity score) [26], and radiological aspects (Balthazar Severity Index) [28,29], and data regarding the evolution and outcome of the patient (length of hospital stay, need for intensive care stay, and mortality) (Table 1).

Among the biological data, the following information was collected: leucocytes, monocytes, neutrophils, hemoglobin (Hb), hematocrit (Ht), alanine aminotransferase (ALT), aspartate aminotransferase (AST), total bilirubin (TB), gamma-glutamyl transpeptidase (GGT), creatinine, BUN, triglycerides, lipase, cholesterol, CRP, NLR (neutrophil count/lymphocyte count), PLR (platelet count/lymphocyte count), MLR (monocyte/lymphocyte count), SII (systemic immune-inflammation index), and SIRI (systemic inflammation response index). All the abovementioned biological data were collected at the time of the patient’s admission. Blood counts were determined using the Sysmex XN-1000 (Sysmex Corporation, Kobe, Japan) and XN-3000 automated hematology analyzers (Sysmex, Etten Leur, The Netherlands). This study was conducted in accordance with the Declaration of Helsinki and was approved by the Ethics Committee of “Dr. Carol Davila” Central Military University Emergency Hospital, No. 509, dated 05 April 2022. Informed consent was obtained from all subjects involved for using and studying the collected data for scientific purposes, and we obtained data from the patients’ medical records.

### 2.2. Statistical Analysis

Qualitative data are expressed as frequencies and percentages, while quantitative data are expressed as medians and interquartile ranges (IQR: 25th percentile–75th percentile). To identify statistically significant differences between the HTGP and AAP groups, comparisons of medians were performed for continuous factors by using the Mann–Whitney U test. At the same time, proportions were analyzed using the chi-squared test or the Fisher exact test. For the statistical analyses, the significance level considered was 0.05, and IBM Statistical Package for the Social Science (SPSS) v.20 was used.

## 3. Results

### Patient Characteristics

A total of 78 patients were included in this study, of which 29 were diagnosed with HTGP, representing 37.18%, and 49 were diagnosed with AAP, representing 62.82%. Males were more frequently affected in both categories. Baseline clinical characteristics are described in Table 2.

The mean age at diagnosis was 44.0 (IQR; 38–54) years in the HTGP group, while in the APP group, it was 49.0 (IQR; 42–57). The HTGP patients were younger, and obesity was more frequent in this group, with 55.17% of the patients being obese. Diabetes was also more frequent in the HTGP patients, with a percentage of 58.62%, while only 22.45% of AAP patients had this comorbidity (*p* < 0.00) (Table 2).

Other comorbidities such as heart failure, arterial hypertension, and chronic renal disease were uncommon in both groups, without statistical significance between the two cohorts. None of the patients presented chronic renal disease.

According to the revised Atlanta severity criteria, 24.14% of patients with HTGP had severe pancreatitis, while 14.29% of the AAP patients met the same criteria (Table 3).

According to the Balthazar index, more severe forms were observed in the HTGP group (*p* = 0.012). For example, 58.62% of patients with HTGP had a Balthazar index of D or greater, while only 34.69% of patients with AAP met this criterion (Table 3).

Regarding the recurrent episodes of AP, patients with hypertriglyceridemia presented with one episode in 13.79% (4/29) of cases, and 10.34% (3/29) of these patients had experienced two previous episodes. The proportion was similar in the alcohol-induced AP group, with 14.29% (7/49) with one AP episode and 10.20% (5/49) with two AP episodes. The recurrent AP with three or more relapses was higher in alcohol-induced AP cases compared with the HTGP group (16.33% vs. 3.45%).

The length of stay was significantly longer for HTGP patients, with a median of 8 (6–16) days, whereas in the AAP group, the median hospitalization stay was 6 (4–10) days (*p* = 0.024). Although ICU admission was twice as common in the HTGP group compared to the AAP group (13.79% vs. 6.12%), the difference was not statistically significant (*p* = 0.414). The difference was also not statistically significant for mortality (*p* = 0.893). Two deaths occurred in the metabolic pancreatitis group, representing 6.90%, and three deaths occurred in the alcohol-induced acute pancreatitis group, representing 6.12%. The organ failure found in the HTGP group was predominantly due to hypoxemia that required correction with supplemental oxygen (4/29), and three patients required ventilatory support. Two cases presented renal failure, one of which was accompanied by cardiovascular distress, and one had sepsis. In the AAP group, four patients presented with respiratory impairment, of whom three required ventilatory support and one had renal failure, also requiring vasopressor support. Another subject had multiple organ failures. Regarding the necrotizing form of AP, in the alcohol-induced pancreatitis group, there were five cases of necrotizing AP, and seven cases in the HTGP group.

In our group, the main indications for surgery were necrosectomy, debridement, and pseudocyst internal drainage with cyst jejunostomy after 8 weeks of disease progression. Since endoscopic necrosectomy is not yet possible in our center, it is performed surgically whenever necessary. Additionally, as we emphasize, the length of hospitalization varied from 0 to 86 days; consequently, some patients developed complications that required surgery or failed to survive. Surgical interventions were not needed in the early stages.

HTGP patients had a median triglyceride level of 2432 (1280–4923) mg/dL and a median cholesterol level of 460 mg/dL (246.5–758). APP patients had a median triglyceride level of 133 (89–177) mg/dL and a median serum cholesterol level of 178.5 (120–216.5) mg/dL (Table 4).

Among inflammatory markers, only fibrinogen was significantly elevated in the HTGP group (739 mg/dL vs. 563 mg/dL) compared to the other group. Other markers like CRP, NLR, PLR, MLR, SII, and SIRI showed no significant differences between AAP and HTGP patients.

New-onset diabetes mellitus was found in 31.05% (9/29) of patients in the HTGP group compared with 2.04% (1/49) of patients in the AAP group (Table 5).

The therapies used for HTGP included fenofibrate, HMG-CoA reductase inhibitors (statins), and omega-3 fatty acids in conjunction with anticoagulants (Table 6).

## 4. Discussion

In our study, we aimed to show the differences in outcomes and severity between hypertriglyceridemia-induced acute pancreatitis and alcoholic acute pancreatitis.

According to our data, the male gender was predominant in both etiologic groups, with HTGP patients being slightly younger and more frequently obese and diabetic than patients in the AAP cohort. These data support similar results from other studies that claim that the typical phenotype of an HTGP patient is represented by a young male patient who is obese or at least overweight [32,33,34].

Men were also more frequently affected by acute alcoholic pancreatitis; this could be because males generally consume more alcohol, although recent epidemiological studies show a tendency toward a narrowing of the gap between genders [35].

Comorbidities like hypertension, cardiac failure, and chronic renal disease showed no statistical significance. The link between hypertriglyceridemia, diabetes, and obesity is expected due to their shared background of metabolic syndrome. However, obesity is independently associated with more severe acute pancreatitis, regardless of etiology [36].

In a study from 2021, Yang et al. proved that both comorbid hypertriglyceridemia and abdominal obesity are independent risk factors for more severe forms and higher incidences of pancreatic necrosis [37].

The literature indicates a higher likelihood of diabetes in both AAP and HTGP patients. Still, no direct studies have been conducted to compare these two etiologies head-to-head [38].

Both alcoholic and hypertriglyceridemic etiologies are risk factors for recurrent pancreatitis, leading to progressive pancreatic damage and, ultimately, diabetes [19].

Diabetes itself can result from acute pancreatitis or contribute to secondary hypertriglyceridemia. Diabetic dyslipidemia is characterized by low levels of HDL, high levels of triglycerides, and mildly elevated or normal LDL-C levels [39,40].

In our study, the Atlanta severity index and Balthazar index demonstrated that HTGP patients experienced more severe forms of acute pancreatitis compared to those with AAP. A higher Balthazar index on a CT scan is associated with a greater extent of pancreatic fluid collection and necrosis. In a retrospective study by Pascual et al., hypertriglyceridemia was found to be positively associated with pancreatic necrosis and peripancreatic fluid collections [41].

According to the revised Atlanta classification, the severity of acute pancreatitis is stratified into mild, moderately severe, and severe categories. Mild acute pancreatitis is defined by the absence of organ failure or local or systemic complications. Moderately severe acute pancreatitis is characterized by transient organ failure or local or systemic complications in the absence of persistent organ failure. Severe acute pancreatitis means there is persistent organ failure [26]. In our retrospective analysis, 82.76% of patients with HTGP had moderate or severe pancreatitis, while in the AAP group, only 36% met the same criteria.

Though only the first two were significant, HTGP patients had more extended hospital stays, higher ICU admission rates, and more significant mortality. One patient in the HTGP group needed a cyst jejunostomy. In another case, the patient had a necrosectomy, abscess debridement, and adhesiolysis, was hospitalized for 86 days, and failed to survive. Neither case involved an early intervention after 8 weeks of hospitalization.

In our group, the primary indications for surgery were necrosectomy, debridement, and internal drainage of pseudocysts with a cyst jejunostomy, rather than in the early stages. Since endoscopic necrosectomy is not possible in our center, it is performed surgically whenever necessary. Our study also had a limited sample size, so further studies with a larger patient population are needed. Also, as we emphasize, the length of hospitalization varied up to 86 days; consequently, some patients developed complications that needed surgery or died. While most studies compare HTGP with non-HTGP pancreatitis, direct comparisons with alcoholic pancreatitis are limited.

In a 2016 retrospective study, Goyal et al. compared HTGP and AAP and found that HTGP patients had more severe forms at admission, as determined by the Atlanta criteria and Balthazar index, with longer hospital stays, higher ICU admission rates, and a greater need for surgery [42]. The literature reports similar findings on HTGP vs. non-HTGP pancreatitis. Shafiq et al. found that HTGP patients are younger, have a higher BMI, greater clinical and radiological severity, and longer hospital stays [43].

A retrospective analysis conducted by He et al. compared HTGP with non-HTGP pancreatitis and revealed higher necrosis and organ failure rates, more extended hospital stay, and higher mortality rates in the HTGP group [44]. Another retrospective study from 2022 by Dancu et al., which analyzed the differences between the HTGP and non-HTGP groups, showed that the HTGP patients were predominantly younger males with higher rates of diabetes and local complications but without a significant difference in terms of hospitalization and mortality rates [34].

Hypertriglyceridemia has been associated with a risk of acute pancreatitis, and high serum levels of triglycerides were shown to be linked to more severe forms and a worse prognosis, regardless of the etiology [45,46].

Since serum levels greater than 1000 mg/dL were the intrinsic diagnostic criterion for HTGP in our study, we expected a difference in TGs between the groups. However, even if alcohol is a recognized secondary cause of hypertriglyceridemia, in our study, the median level of triglycerides in patients in the AAP group was only slightly higher than the upper limit. One possible explanation is that both the pattern, mode, and duration of alcohol consumption can influence the way the lipid profile is altered [9,10]. All these variables were not considered when the data were collected.

There is a concern that acute pancreatitis might obscure an underlying pancreatic cancer, making it harder to detect through imaging. Recent studies suggest that when pancreatic cancer is diagnosed within 90 days of acute pancreatitis, it tends to be at an earlier stage, with higher chances of surgical resection and improved survival rates. Therefore, timely cancer screening in these patients could enhance survival outcomes [47].

The risk of pancreatic cancer rises sharply following an acute pancreatitis diagnosis, gradually declines after two years, and remains elevated for up to a decade. Additional research is needed to clarify the long-term impact of acute pancreatitis on pancreatic cancer risk [48,49,50].

Regarding the inflammatory state, various inflammatory markers have emerged as potential baseline indicators for acute pancreatitis severity. Systemic inflammation markers have been studied intensively in the last few years, and they are correlated with a more severe course of the disease and higher persistent organ failure rates [51].

In our analysis, fibrinogen was the only inflammatory marker showing a significant difference between the two groups. All other inflammation biomarkers, including CRP and systemic inflammation indices (CRP, NLR, PLR, MLR, SII, and SIRI), had no discriminative value when comparing the two etiologies. Several studies have investigated the predictive value of these markers in determining the etiology of acute pancreatitis. In a 2017 study, Wang et al. showed that in hypertriglyceridemia-induced acute pancreatitis, the NLR predicted more severe forms and higher organ failure rates on admission, with higher rates of complications, systemic inflammatory response syndrome, and acute kidney injury [52]. The latest studies highlighted that ferritin could represent a possible marker related to all causes of acute pancreatitis severity, but no study presented results in hypertriglyceridemia or alcohol-induced pancreatitis [53].

Another study from 2022 by Lu et al. outlined that in hypertriglyceridemia-induced acute pancreatitis, there is a significant association between a high NLR on admission and an increased rate of persistent organ failure [54].

Although CRP is a well-studied predictor of a more severe and complicated course of acute pancreatitis, our study still found no difference between groups. One possible explanation is that HTGP patients might have a history of dyslipidemia and were already receiving lipid-lowering treatment, which was continued during hospitalization. However, despite being on such therapy, most patients in the HTGP group did not adhere to the dietary recommendations. These patients experienced acute pancreatitis following the consumption of high-fat meals. Studies have shown that both statins and fibrates lower CRP, and the effect of lowering CRP is independent of the reduction in LDL cholesterol [55,56]. We also observed lower serum sodium levels in patients with hypertriglyceridemia-induced acute pancreatitis. This was due to pseudohyponatremia, a well-known phenomenon characterized by the replacement of water with lipids within the serum. A study by Wang et al. from 2019 showed that it could be a helpful clue in differentiating hypertriglyceridemia from other etiologies of acute pancreatitis, preventing possible delays in diagnosis [57]. Acknowledging the presence of pseudohyponatremia in HTGP has clinical implications because sodium levels do not require correction in this situation; correcting triglyceride levels will also lead to a gradual correction of sodium levels [58].

To our knowledge, no studies have independently evaluated the usefulness of these markers in alcohol-induced acute pancreatitis. Most of the results come from studies that compare acute alcoholic pancreatitis with etiologies other than HTGP. NLR proved to be a good predictor for assessing severity in acute biliary pancreatitis compared to acute alcoholic pancreatitis [59].

This study has several limitations. First, it is a retrospective single-center study; data were extracted using disease codes from patients’ medical records, and values such as hemoglobin A1C (HbA1C) levels were unavailable. Second, the sample size of patients included was small, focusing on HTGP and AAP cases, which may not provide robust statistical power for our results and limit the ability to establish causation. There is limited data regarding specific timelines for the recurrence of previous episodes in patients with a history of recurrence. Despite these limitations, this study highlights the differences between acute pancreatitis cases and offers insights that may serve as a foundation for future research. Further prospective studies with a larger cohort of patients are needed to confirm and extend our results.

We emphasize that most studies in the literature compare pancreatitis induced by hypertriglyceridemia with non-hypertriglyceridemia-induced pancreatitis without accounting for the fact that the latter represents a heterogeneous group of etiologies encompassing acute pancreatitis.

## 5. Conclusions

The pattern of acute pancreatitis is related to etiology and might have different grades of severity. In our study, we found that hypertriglyceridemia-induced pancreatitis resulted in twice as many admissions to the intensive care unit, and more than 80% of patients had moderate or severe acute pancreatitis compared to those with alcohol-induced pancreatitis.

## Figures and Tables

**Table 1:** Balthazar score, necrosis scoring system, and the revised Atlanta classification of acute pancreatitis [26,27,28,29,30,31].

Balthazar Score	Necrosis Score	The Revised Atlanta Classification	Atlanta Diagnosis Criteria (Minimum Two Criteria for Diagnosis)
A- Unaltered pancreatic appearance	None (0 pts.)	MAP Absence of organ failure Absence of local or systemic complications	Abdominal pain suggestive of pancreatitis
B- Localized or extensive pancreas enlargement	≤30% (2 pts.)		
C- Peripancreatic inflammatory changes	>30–50% (4 pts.)	MSAP Transient (<48 h) organ failure and/or local or systemic complications with transient organ failure	Serum amylase or lipase level at least ×3 upper normal value
D- Single peripancreatic fluid collection	>50% (6 pts.)		
E- More than one fluid peripancreatic collection or presence of retroperitoneal air		SAP Persistent organ failure (>48 h) Single or multiple organ failure	Characteristic features of image studies

MAP—mild acute pancreatitis, MSAP—moderately severe acute pancreatitis, SAP—severe acute pancreatitis.

**Table 2:** Baseline clinical characteristics.

Characteristics	HTGP (*N* = 29)	AAP (*N* = 49)	*p*-Value
Age, median, years (IQR)	44.00 (38–54)	49.00 (42–57)	0.105
Sex			
Female	13.79% (4/29)	4.08% (2/49)	
Male	86.21% (25/29)	95.92% (47/49)	
Obesity	55.17% (16/29)	16.33% (8/49)	0.000
Diabetes mellitus	58.62% (17/29)	22.45% (11/49)	0.001
Arterial hypertension	41.38% (12/29)	40.82% (20/49)	0.961
Heart failure	0% (0/29)	8.16% (4/49)	0.114
Median hospitalization (days, IQR)	8 (6–16)	6 (4–10)	0.024
ICU admission	13.79% (4/29)	6.12% (3/49)	0.414
Mortality	6.90% (2/29)	6.12% (3/49)	0.893
Need for surgery	6.90% (2/29)	0% (0/49)	
Cigarette smoking habit	41.38% (12/29)	44.90% (22/49)	0.762
High-fat diet	37.93% (11/29)	18.37 (9/49)	0.056

ICU—intensive care unit, IQR—interquartile range, HTGP—hypertriglyceridemia-induced acute pancreatitis, AAP—alcohol-induced acute pancreatitis.

**Table 3:** Severity of pancreatitis as per Atlanta criteria and Balthazar index.

	HTGP	AAP	*p* Value
Atlanta criteria			
Mild acute pancreatitis	5 (17.24%)	26 (53.06%)	0.008
Moderately acute pancreatitis	17 (58.62%)	16 (32.65%)	
Severe acute pancreatitis	7 (24.14%)	7 (14.29%)	
Balthazar index			
B	2 (6.90%)	16 (32.65%)	
C	10 (34.48%)	16 (32.65%)	
D	11 (37.93%)	15 (30.61%)	
E	6 (20.69%)	2 (4.08%)	

HTGP—hypertriglyceridemia-induced acute pancreatitis, AAP—alcohol-induced acute pancreatitis.

**Table 4:** Laboratory characteristics.

Laboratory Data (Median Range, Min; Max)	HTGP	AAP	*p* Value
Lipase level, median (IQR)	359 (187–694)	379.5 (176.5–1295)	0.652
TGs, median (mg/dL)	2432 (1280.5–4923)	133 (89–177)	0.000
Cholesterol (mg/dL)	460 (246.5–758)	178.5 (120–216.5)	0.000
Hemoglobin (g/dL)	15.6 (14.7–17.5)	14.6 (13.2–16.0)	0.016
Hematocrit (%)	42 (38.5–45.6)	41.6 (38.2–44.5)	0.538
CRP	120.1 (72–199)	101.74 (14.33–280.15)	0.211
Fibrinogen (mg/dL)	739.0 (573–1052)	563.5 (379–844)	0.030
Na (mmol/L)	130 (125–133)	137 (134–139)	0.000
Urea (mg/dL)	26.0 (20–36)	36.0 (25–45)	0.051
Creatinine (mg/dL)	0.86 (0.65–1.25)	0.82 (0.70–0.99)	0.501
ALT (UI/L)	36 (24–59)	37 (23–95)	0.466
AST (UI/L)	44 (31–84)	52 (25–94)	0.951
GGT (UI/L)	103 (47.95–404)	164 (70–401)	0.418
Total bilirubin (mg/dL)	0.88 (0.64–1.34)	0.77 (0.49–1.71)	0.707
Serum total calcium	8.59 (7.88–8.83)	8.91 (8.45–9.31)	0.033
CA19-9	27.89 (15.72–41.03)	26.82 (5.73–262.03)	0.867
NLR	6.71 (4.31–9.68)	5.51 (3.69–10.29)	0.308
PLR	136.92 (92.49–207.78)	142.66 (102.99–207.83)	0.698
MLR	0.55 (0.35–0.78)	0.52 (0.37–0.77)	0.897
SII	1302.6 (966.68–2368.67)	1345 (713.75–2561.18)	0.955
SIRI	5.76 (3.24–10.55)	5.36 (2.72–8.71)	0.344

TGs—serum triglycerides, CRP—C-reactive protein, ALT—alanine transaminase, AST—aspartate aminotransferase, GGT—gamma-glutamyl transpeptidase, CA19-9—carbohydrate antigen 19-9, NLR—neutrophil count/lymphocyte count, PLR—platelet count/lymphocyte count, MLR—monocyte/lymphocyte count, SII—systemic immune-inflammation index, SIRI—systemic inflammation response index. Normal values: lipase, 3–67 U/L; TGs, 30–150 mg/dL; cholesterol, <200 mg/dL; hemoglobin, 13.2–16.6 g/dL; Ht, 40–52%; CRP, <5 mg/L; fibrinogen, 276–471 mg/dL; Na (serum sodium), 136–145 mmol/L; urea, 19–44 mg/dL; creatinine, 0.9–1.2 mg/dL; AST, 11–34 U/L; ALT, <45 U/L; total bilirubin, <0.2 mg/dL; GGT, 5.00–55.00 U/L; serum total calcium, 8.4–10.2 mg/dL; CA19-9, <37 U/mL.

**Table 5:** Diabetes mellitus (DM) status in HTGP and AAP groups.

	HTGP (Cases)	AAP (Cases)	*p*-Value
New-onset DM	31.03% (9/29)	2.04% (1/49)	0.001
Non-DM	44.83% (13/29)	77.55 (38/49)	
Previously diagnosed DM	24.14 (7/29)	20.41 (10/49)	
Oral antidiabetic agents	10.34% (3/29)	12.245 (6/49)	0.800
Rapidly acting insulin	75.86% (22/29)	2.04% (1/49)	0.000
Long-acting insulin	44.83% (13/29)	0.00% (0/49)	0.000

**Table 6:** Medical therapy in the cohort study.

Medical Therapy	HTGP (Cases)	AAP (Cases)	*p*-Value
Fenofibrate, 145 mg po	27.59% (8/29)	2.04% (1/49)	0.001
Fenofibrate, 160 mg po	62.07% (18/29)	2.04% (1/49)	0.000
HMG-CoA reductase inhibitors (statins)	68.96% (20/29)	0.00% (0/49)	
Omega-3 fatty acids	65.52% (19/29)	0.00% (0/49)	
Previous lipid-lowering treatment	31.03% (9/29)	4.08% (2/49)	0.001
Low-molecular-weight heparin	37.93% (11/29)	24.49% (12/49)	0.208
Unfractionated heparin	41.38% (12/29)	2.04% (1/29)	0.000

## Data Availability

The data are available upon reasonable request.
